# Acute Hydronephrosis Secondary to Methadone-Induced Constipation

**DOI:** 10.7759/cureus.25090

**Published:** 2022-05-17

**Authors:** Sameer Kandhi, Siddharth Chinta, Ana P Urena Neme, Michael Victoria Guerrero, Miguel A Rodriguez Guerra

**Affiliations:** 1 Internal Medicine, BronxCare Health System, Bronx, USA; 2 Cardiology, Medicina Cardiovascular Asociada, Santo Domingo, DOM; 3 Medicine, Instituto Tecnologico de Santo Domingo, Santo Domingo, DOM; 4 Medicine, Montefiore Medical Center, Wakefield Campus, Bronx, USA

**Keywords:** hydronephrosis, laxatives, opioid use disorders, methadone, opioid induced constipation

## Abstract

Opioid-induced constipation is a significant medical problem accounting for over 40% to 60% of patients without cancer receiving opioids. We report a unique case of a 71-year-old male with a history of opioid use disorder now on methadone maintenance presenting with severe opioid-induced constipation and fecal impaction causing extrinsic compression on the right-sided ureter resulting in right hydronephrosis and hydroureter that improved with aggressive bowel regime with the stool softener, laxatives and enemas.

Methadone alone can predispose to hydroureter with hydronephrosis due to external compression from the severe intestinal dilation secondary to opioid-induced constipation.

## Introduction

Opioids are commonly prescribed analgesics for acute or chronic pain. Their use has increased in the past years, and the efforts to reduce their consumption are because of their addictive nature. Their side effects include lethargy, nausea, CNS depression, pruritus, and constipation [1]. While opioid effectiveness in treating pain is high, 18.9% of patients discontinued their use due to significantly worsening quality of life-related opioid side effects [2].

Addiction to these medications has been a burden to the healthcare and criminal justice system of the United States [3]. The deescalation of opioid therapy is a challenge due to the pain control they provide and the need to minimize withdrawal symptoms [4]. This addiction requires both medical treatment and psychological treatment to cease its use. Research has found that medications for opioid use disorder in addition to an opioid receptor agonist (methadone), partial agonist (buprenorphine), or opioid antagonist (extended-release naltrexone) can facilitate recovery [5]. Methadone, a synthetic opioid, is a mu-opioid receptor agonist and NMDA receptor antagonist. Unlike other opioids, it possesses a longer half-life and fewer withdrawal symptoms [4]. This long-acting medication is used for moderate to severe pain non-responsive to non-narcotic drugs, detoxification, treatment of opioid use disorder, and treatment of neonatal abstinence syndrome [6]. Multiple challenges to the discontinuation of Methadone therapy exist. The first one is the limited evidence on optimal treatment duration, although studies show that tapering and discontinuation of this therapy lead to high rates of relapse and increased risk of death [7]. 

Here we present a case of a patient on methadone who presented with abdominal pain with severe constipation and was found to have hydronephrosis and hydroureter due to fecal compression.

## Case presentation

We report a case of a 71-year-old male with a past medical history of opiate dependence, currently on methadone maintenance (85mg daily) for the past year; he presented to our emergency room with persistently progressive diffused abdominal pain for three days, it is 6/10 intensity, associated with constipation for the same period and one period of fecal incontinence. On further inquiry, the patient-reported of altered bowel habits with episodes of diarrhea alternating with severe constipation for the last two to three months. The patient also stated that he had an episode of fecal impaction, requiring recurrent visits to multiple emergencies. His last bowel movement (BM) was three days ago. He denied symptoms of fever, melena, hematochezia, nausea, vomiting, weight loss, lack of appetite, burning micturition, changes in urinary frequency/urgency, hematuria, or urinary habit changes. The patient denied any other significant medical co-morbidities, past surgical, family history, tobacco, alcohol, or current use of recreational drugs. His only home medication happens to be methadone 85mg, with the last dose being on the day of admission.

In the emergency department, the patient was found to be afebrile and vitally stable. Physical Examination was significant for distended abdomen with a tympanic note to percussion, generalized tenderness in all quadrants of the abdomen, and decreased bowel sounds on auscultation, without palpable masses. His initial labs were significant for only mild normocytic normochromic anemia and normal thyroid function level (Table 1).

**Table 1:** Initial Laboratory test results

Lab Tests	On Admission	Reference
WBC count	7.2	4.8-10.8 k/uL
RBC Count	3.85	4.50-5.90 MIL/uL
HGB	11.0	12.0-16.0 g/dL
Hematocrit	34.9	42-51 %
MCV	90.6	80-100 fL
Platelet	204	150-400 k/uL
Sodium	141	135-145 mEq/L
Potassium	3.5	3.5-5.0 mEq/L
Chloride	105	98-108 mEq/L
Bicarbonate	26	24-30 mEq/L
Calcium	9	8.5-10.5 mEq/L
Magnesium	2.1	1.7-2.2 mg/dL
Phosphorous	3.3	3.4-4.5 mg/dL
Blood Urea Nitrogen	23	8-26 mg/dL
Creatinine	1.4	0.5-1.5 mg/dL
Total Bilirubin	0.4	0.2-1.1 mg/dL
Direct Bilirubin	0.2	0.0-0.3 mg/dL
Alkaline Phosphatase	57	56-155 unit/L
Aspartate Transaminase	24	9-48 unit/L
Alanine Aminotransferase	10	5-40 unit/L
TSH	3.8	0.5-5.0 mIU/L

**Figure 1:**
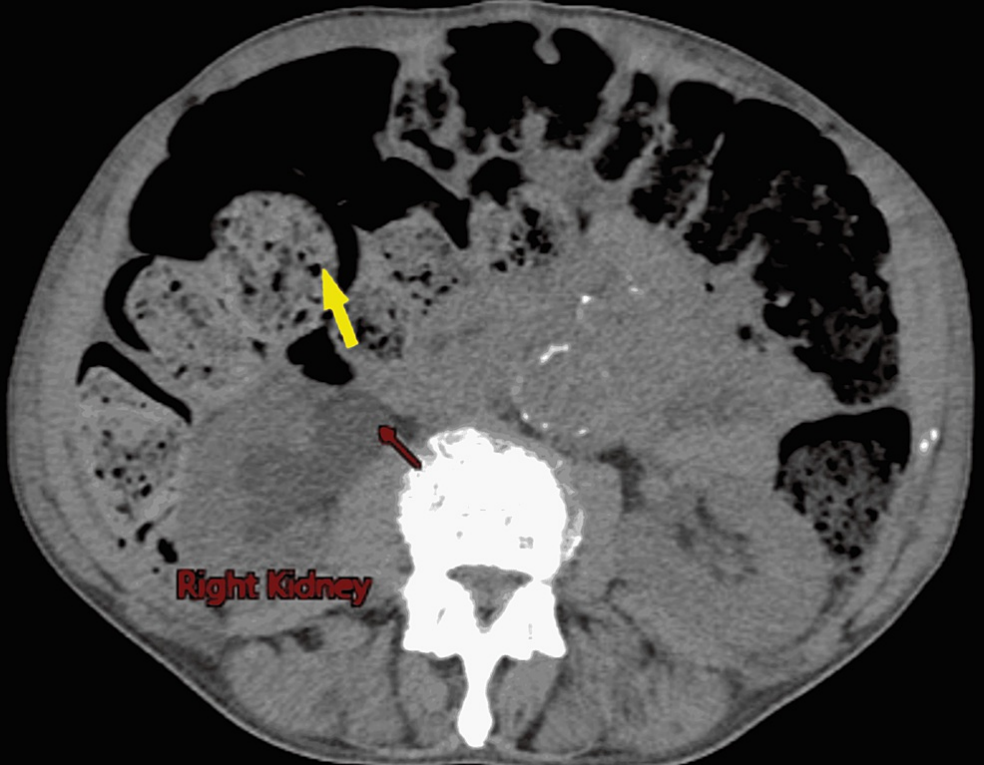
Computerized tomographic image of the patient’s abdomen (cross-sectional view) showing the right-sided hydronephrosis (red arrow) along with severe constipation with bowel dilation (yellow arrow)

**Figure 2:**
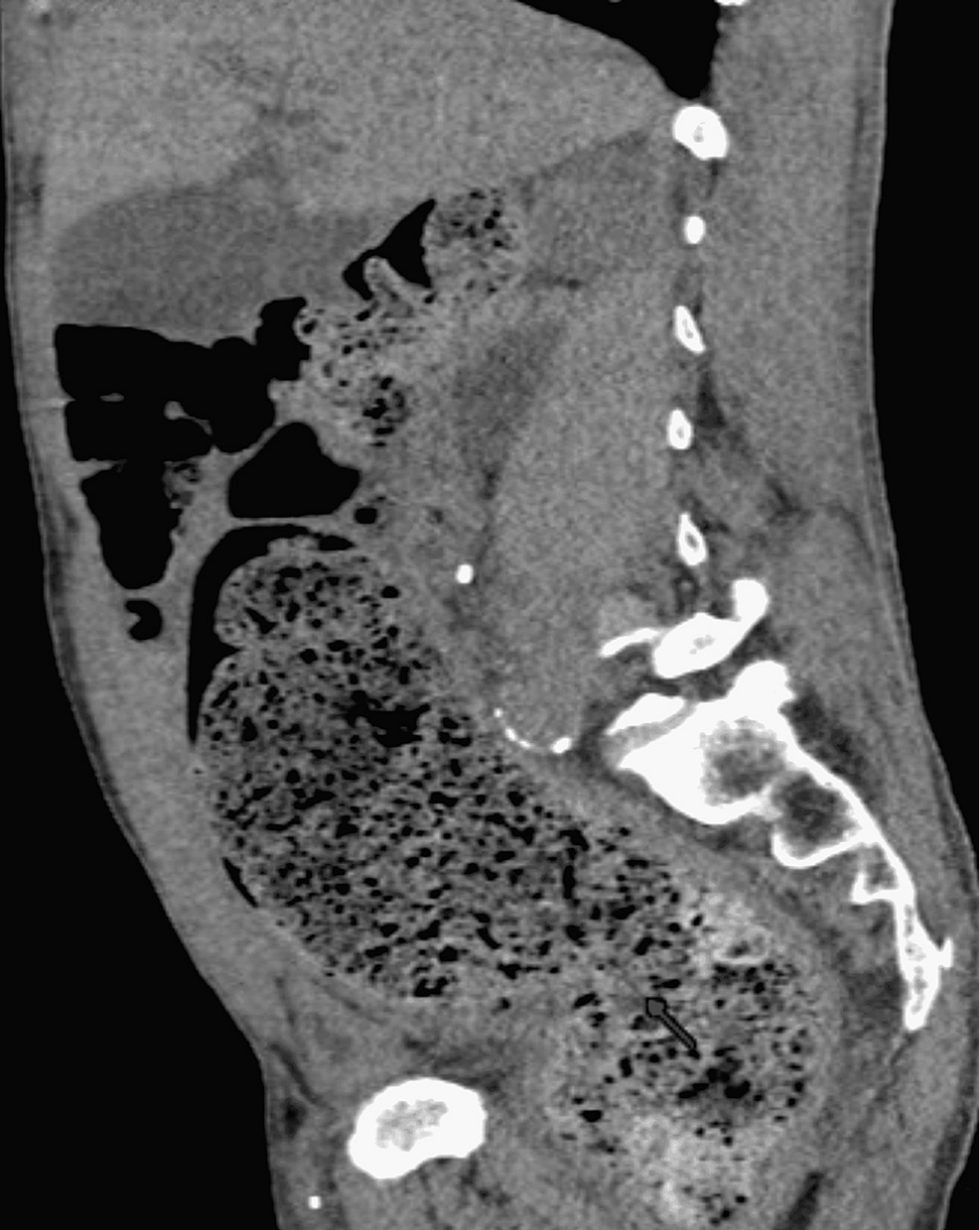
Computerized tomographic image of abdomen (sagittal view) showing the large stool burden in sigmoid colon and rectum (red arrow)

**Figure 3:**
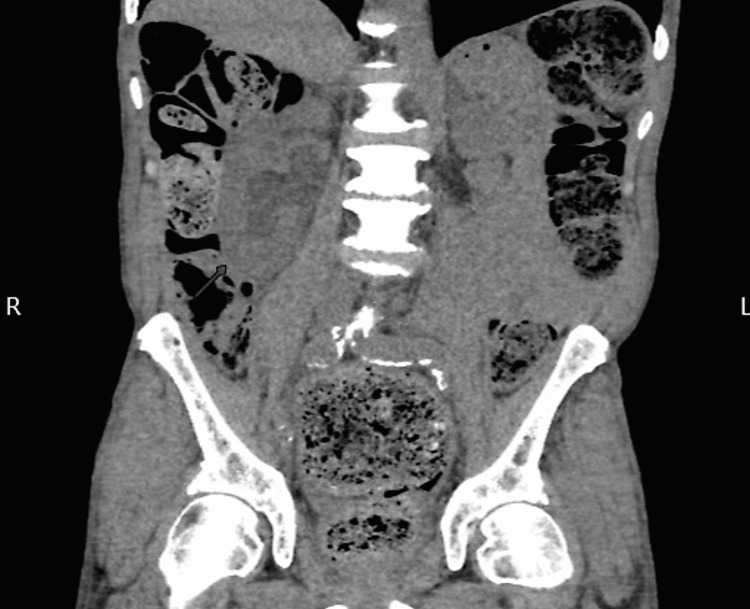
Another computerized tomographic images of abdomen (coronal view) showing severe sigmoid dilation due to constipation on pelvic area with bladder collapse

**Figure 4:**
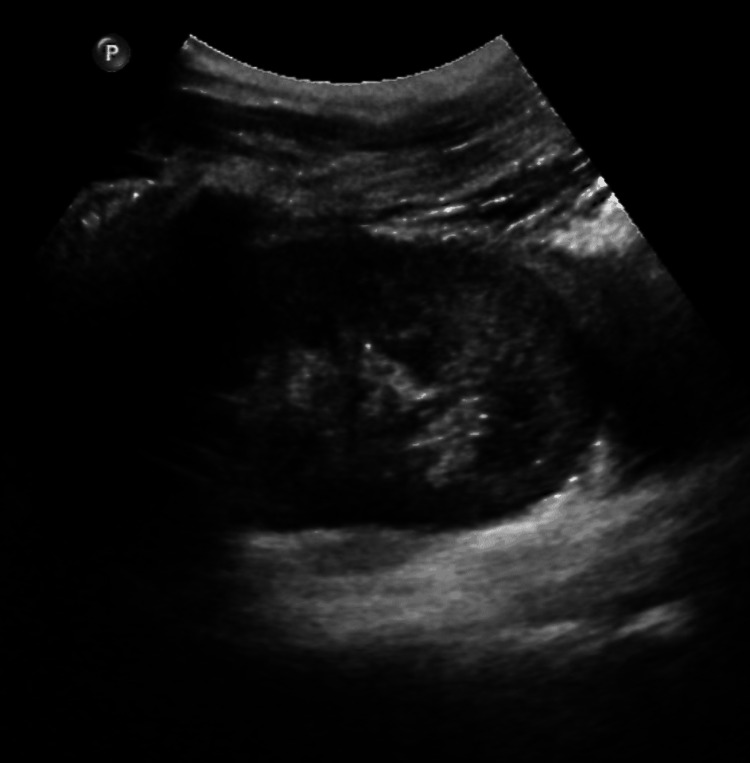
Ultrasonogram of the right kidney post therapy showing resolved hydronephrosis

Computerized tomographic imaging (CAT scan) of the abdomen and pelvis showed severe fecal retention with associated significant bowel dilation, a large right-sided hydroureter with hydronephrosis (Figures 1-3). The right-sided hydronephrosis and hydroureter were secondary to extensive stool burden in the distal colon, causing extrinsic compression on the ureter. The patient underwent manual fecal disimpaction along with soap suds and fleet enemas while still in the emergency and was then admitted to the medical floor eventually for management of his severe constipation. While on the floor, the patient refused to be weaned of his methadone dose, he was started on an aggressive bowel regimen involving polyethylene glycol 17g qd, senna 30mg hs, docusate 200mg q12h, and serial mineral oil enemas. Then the patient had a large BM, and continue to have 2-3 BM a day. On day 3, the patient abdominal pain and distention improved. A kidney and bladder ultrasound showed normal right kidney and ureter with no hydronephrosis or hydroureter (Figure 4). The patient was discharged asymptomatic with regular BM, to be followed by his primary doctor.

## Discussion

The Rome IV criteria define opioid-induced constipation (OIC) as new-onset or worsening constipation at the initiation or change of opioid therapy. In addition, the patient must present two or more symptoms of straining, hard stools, tenesmus, anorectal blockage, the need for manual maneuvers to defecate, or less than three BMs per week [7]. The incidence of OIC varies substantially from 15% to 81% [3]. It also accounts for over 40% to 60% of patients without cancer receiving opioids. Risk factors that predispose patients to developing OIC are advanced age, female gender, reduced mobility, hypercalcemia, altered nutritional intake, and anal fissures [8].

The most common symptoms of opioid-induced constipation are abdominal discomfort, nausea, gas, decreased appetite, reflux, bloating, straining, incomplete evacuation, and hard BMs [9]. In addition, it is essential to know that patients do not develop tolerance to constipation [8]. The binding of opioids causes these side effects on the μ and δ receptors located across the gastrointestinal system in the myenteric and submucosal neurons, which results in reduced neuronal activity and neurotransmitter release [2]. Therefore, leading to increased anal sphincter tone, inhibition of water and electrolyte secretions, decreased peristalsis, increased non-propulsive contractions, and decreased rectal sensitivity [9]. Also, opioids increase water absorption via prolonged stasis, which causes dried-up feces and straining difficulties [10]. Moreover, the increased anal sphincter tone further contributes to difficulties in the initiation of defecation [2].

In Lugoboni et al. study from 2016, a high prevalence of constipation and reduced quality of life (QoL) was found among patients treated with methadone. Similarly, Mattick et al. reported that the prevalence of constipation in patients utilizing methadone was 14% [11]. Furthermore, in Habe et al. in 2017, the primary symptoms reported with methadone were constipation, sweating, nausea or vomiting, insomnia, drowsiness, and sexual difficulties in about 8%-20% of patients [12]. Compared to non-opioid users, methadone-dependent patients also had a significantly higher rate of retained solid stool in the study by Verma et al. in 2012 [13].

A significant complication of the OIC is fecaloma. This is a mass of inspissated stool that results from the accumulation of feces in the rectum or rectosigmoid colon. They may cause distension and increased pressure on the colonic wall. The increased intraluminal pressure may lead to ulceration, localized ischemic necrosis of the colonic wall, and eventual perforation [14]. The fecaloma can lead to acute urinary tract obstruction with extrinsic ureteral compression due to the anterior displacement of the bladder base induced by the dilatation of the rectosigmoid colon [15]. Acute urinary tract obstruction by giant fecaloma is rare, but cases have been reported.

Other complications of chronic constipation include abdominal compartment syndrome (ACS), defined as organ dysfunction caused by an increase in intra-abdominal pressure greater than 20 millimeters of mercury. The World Society of the Abdominal Compartment Syndrome (WSACS) classifies this condition by its underlying cause: decreased abdominal compliance (e.g., burns); increased intra-abdominal contents (e.g., hemoperitoneum, ascites); increased intraluminal contents (e.g., intestinal volvulus, ileus, and constipation), capillary leak/fluid resuscitation, and miscellaneous causes such as obesity and peritonitis [16].

The first step of treatment for OIC involves preventive measures such as increasing water and fiber intake [17]. However, severe symptomatic cases of OIC requiring medical attention are usually managed using a laxative. The choice of agent and the dosing are empiric - with most cases responding to osmotic agents (polyethylene glycol) or stool stimulants (bisacodyl/senna) over stool softeners (docusate). However, there are no adequately powered randomized trials comparing sennosides, docusate, lactulose, or PEG. Patients with suspected fecaloma or fecal impactions secondary to severe constipation have significantly improved with manual fecal disimpaction and rectal enemas (mineral oil enema, irritant enema). Other approved therapies for OIC include methylnaltrexone, naloxone, naldemedine, and lubiprostone. These agents are reserved for only severe refractory cases of OIC that have not responded to initial laxative therapy/enema therapy or manual disimpaction [18-20].

We did not find reported literature on hydronephrosis caused by fecalomas secondary to methadone use. Our patient is a unique case of reversible overflow hydronephrosis with hydroureter due to methadone-induced constipation with fecal impaction.

## Conclusions

Severe constipation secondary to maintenance methadone dose alone can predispose to hydroureter with hydronephrosis. This external obstructive disorder could lead to renal failure or a severe intestinal dilation to a bowel rupture; this is why an early approach is fundamental to avoid potential complications. These patients need a close follow-up and an intense reinforced education about opioids and methadone side effects.
